# Emission characteristics of diethylhexyl phthalate (DEHP) from building materials determined using a passive flux sampler and micro-chamber

**DOI:** 10.1371/journal.pone.0222557

**Published:** 2019-09-20

**Authors:** Naohide Shinohara, Atsushi Mizukoshi, Mayumi Uchiyama, Hirofumi Tanaka

**Affiliations:** 1 Research Institute of Science for Safety and Sustainability (RISS), National Institute of Advanced Industrial Science and Technology (AIST), Tsukuba, Ibaraki, Japan; 2 Department of Environmental Medicine and Behavioral Science, Kindai University Faculty of Medicine, Osakasayama, Osaka, Japan; 3 MC Evolve Technologies Corporation, Inashiki, Ibaraki, Japan; University of Miami, UNITED STATES

## Abstract

Emission rates of diethylhexyl phthalate (DEHP) from building materials, such as vinyl floorings and wall paper, determined using a passive flux sampler (PFS) were constant over the week-long measurement period. Emission rates for vinyl floorings and wallpaper were linearly correlated to the inverse of diffusion distance, which corresponds to the internal depth of the PFS. Surface-air DEHP concentrations (*y*_0_) were estimated as 1.3–2.3 μg/m^3^ for materials having a boundary layer molecular diffusion rate-limiting step. The partition coefficient (*K*_material-air_) was estimated as 3.3–7.5 × 10^10^ for these materials. Additionally, emission rates of DEHP from same building materials determined using a micro-chamber were 4.5–6.1 μg/m^2^/h. Mass transfer coefficients in the micro-chamber (*h*_m_) were estimated by comparing the results using the PFS and micro-chamber, and these were 1.1–1.2 × 10^−3^ and 8.1 × 10^−4^ m/s for vinyl floorings (smooth surface) and wallpaper (rough surface), respectively. The thickness of boundary layer on the surface of building materials in the micro-chamber were estimated to be 2.5–2.6 and 3.7 mm for vinyl floorings and wallpaper, respectively.

## Introduction

Diethylhexyl phthalate (DEHP) is widely used as a plasticizer in many plastics such as polyvinyl chloride (PVC); as a consequence, DEHP can be found in numerous consumer products such as vinyl flooring, wallpaper, vehicle interiors, and toys [1]. Since DEHP molecules are not chemically bound to the PVC [2], and since the vapor pressure of DEHP is low, DEHP is slowly emitted from PVC products to the surrounding environment [3] and often detected in indoor environments [4, 5]. In terms of human toxicity, DEHP is known to induce some adverse health effects such as reproductive toxicity [6, 7], asthma, and allergies [8, 9].

**Fig 1:**
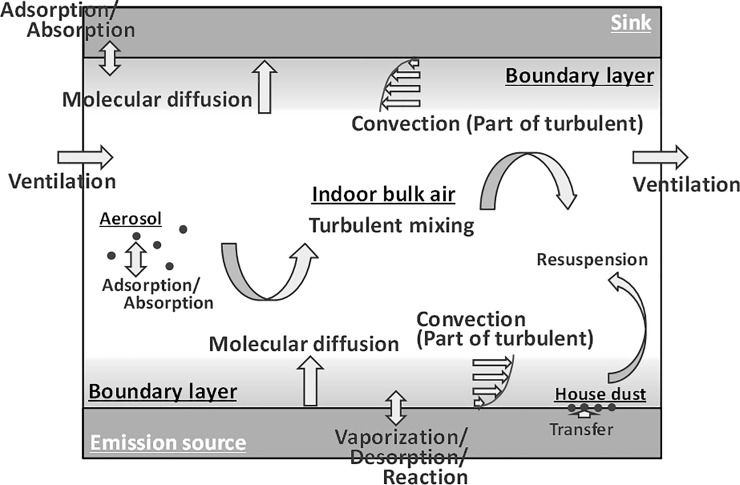
Schematic representation of semi-volatile organic compounds (SVOCs) in an indoor environment.

The behaviors of DEHP in an indoor environment are shown in Fig 1 and S1 Fig. Owing to its low vapor pressure, DEHP, once emitted into an indoor environment, is easily adsorbed onto the wall, floor, and other interior surfaces and particles [10]. However, the amount of DEHP transferred from known emission sources to an indoor environment, as well as the amounts adsorbed on indoor surfaces and particles, is less well known. For example, phthalate exposure levels are significantly higher in infants with PVC flooring in their bedrooms than those without [11]. To decrease human exposure to DEHP in indoor environments, a deeper knowledge regarding the emission of DEHP from several indoor sources is important. Although the emission chamber method is often used to determine the emission rates of volatile organic compounds (VOCs) from materials, DEHP emission rates are more difficult to determine by this method owing to its low vapor pressure and strong sorption. Therefore, several methods have been proposed and used to obtain the emission characteristics of DEHP from PVC materials; these methods include CLIMPAQ (Chamber for Laboratory Investigations of Materials, Pollution and Air Quality) [12, 13], the sandwich chamber [2], improved sandwich chamber [14], FLEC (Field and Laboratory Emission Cell) [12, 13, 15], the passive flux sampler (PFS) [16], and micro-chamber methods[17–19], thermal desorption tube method [20], C_m_-history method [21], early stage C-history method [22], and SPME-based method [23, 24]. Although valuable information on emission can be obtained by using the PFS at multiple diffusion distances, these data were not obtained in previous studies [16].

The objectives of this study were evaluation of the DEHP emission parameters on the emission surface in the indoor environment/in-chamber in by using PFS at multiple diffusion distances. For the purpose, the emission rates of phthalate esters from building materials such as flooring and wallpaper were measured using the PFS method at multiple diffusion distances; surface concentrations of DEHP on the emitting materials were estimated based on the PFS measurement results. In addition, the emission rates from building materials were also measured using the micro-chamber method; the boundary layer thicknesses and mass-transfer coefficients in a micro-chamber apparatus were estimated based on PFS and micro-chamber measurement results. We also determined the DEHP contents in these building materials to obtain the partition coefficient.

## Materials and methods

### Passive flux sampler

**Fig 2:**
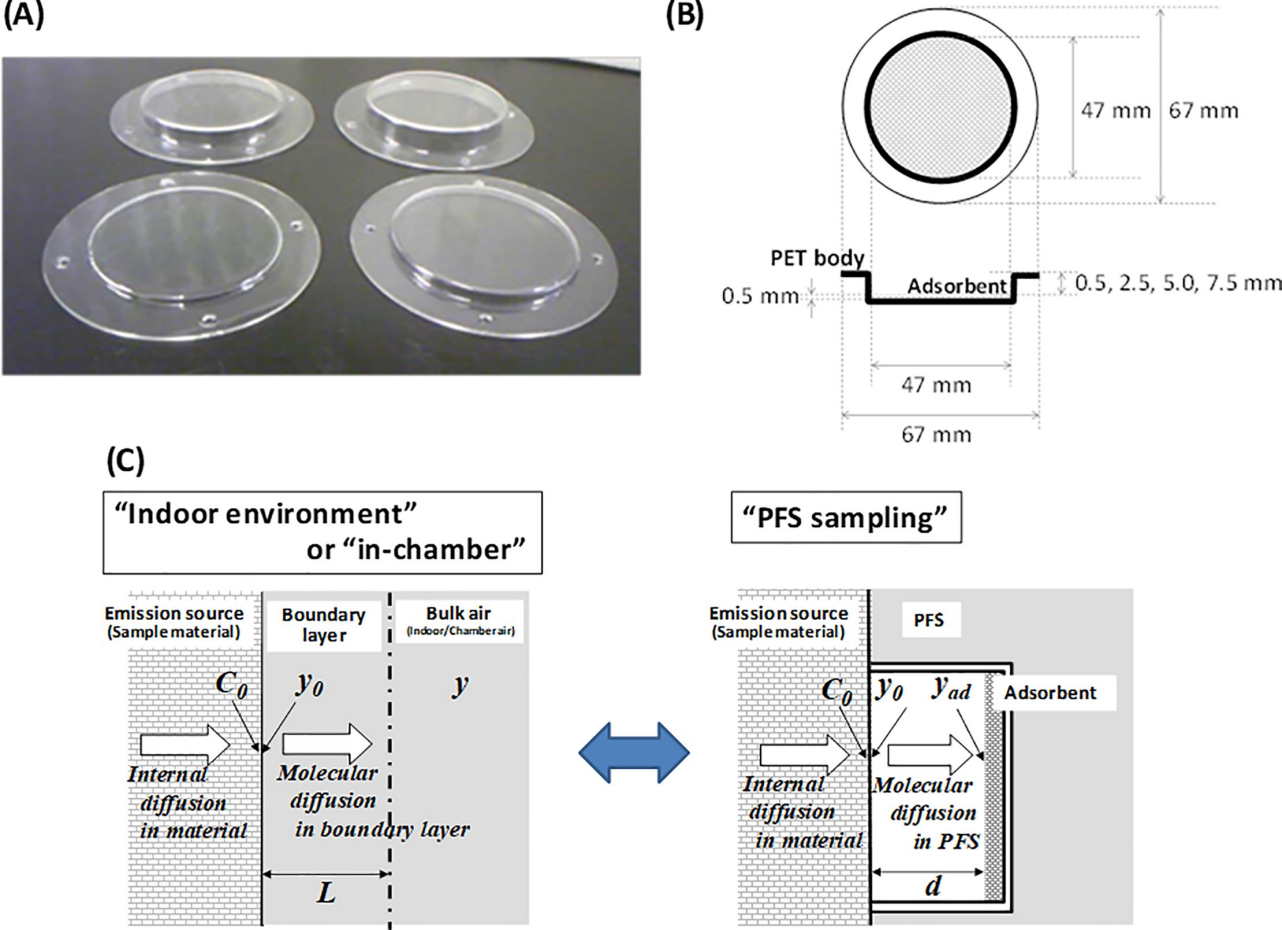
(A) A photograph of the PFS, (B) diagram illustrating the design of the PFS, and (C) diagram illustrating the mechanism of the PFS. The diameter of the diffusion area in the PFS is 47 mm. The diffusion distances in the PFS are 0.5, 2.5, 5.0, and 7.5 mm. In an actual indoor environment or in a micro-chamber, DEHP diffuses from inside to the surface of the material, diffuses in the gas-phase boundary layer, and mixes in the bulk air. DEHP behavior in the boundary layer can be reproduced in the PFS. In the Figure, *C*_0_ is the surface concentration of DEHP in the building material [μg/m^3^], *y*_0_ is the surface-air concentration of DEHP on the building material [μg/m^3^], *y* is indoor concentration [μg/m^3^], *y*_ad_ is the adsorbent surface-air concentration of DEHP [μg/m^3^], *L* is the thickness of boundary layer on the emission source in the indoor environment [m], and *d* is the diffusion distance [m].

A PFS (Fig 2(A) and 2(B), produced in National Institute of Industrial Science and Technology (AIST)) was used to measure the emission rates of phthalate esters from building materials. When the PFS was placed on the sample material, DEHP emitted from sample material was molecularly diffused inside PFS and adsorbed onto the adsorbent. Internal diameter of PFS is 47 mm and depth of PFS after setting the adsorbent (diffusion distance) are 0.5, 2.5, 5.0, and 7.5 mm, respectively (Table 1).

**Table 1:** Test condition for micro-chamber and PFS.

	Micro-chamber	PFS
Temperature [°C]	28 ± 1	28 ± 1
Relative humidity [%]	50	-
Volume [L]	0.63	0.00087–0.013
Air flow rate [mL/min]	0.015	-
Air exchange rate [/h]	1.4	-
Area of test piease [m^2^]	0.0053	0.0017
Internal surface area [m^2^]	0.037	0.000074–0.0011
Chamber surface material	Silane-treated glass	PET

A schematic illustrating the mechanism of PFS sampling to obtain the emission flux from emission source is shown in Fig 2(C) and S2 Fig. In an actual indoor environment or in a micro-chamber, chemicals diffuse from the inside to the surface of the material, volatilize/desorb at the surface of material, diffuse in the gas-phase boundary layer from the surface of the material to bulk indoor/in-chamber air, and mix in the bulk indoor/in-chamber air (Fig 2(C)). The behavior on the surface of the material in the actual environment/in-chamber can be reproduced in the PFS (Fig 2(C)). When the emission flux from the source material depends on Fick’s law, the rate-limiting step of DEHP emission was diffusion in the gas-phase boundary layer. In this case, the demand for DEHP is insufficient compared to the supply capacity of DEHP from the inside of the source material to the surface. Therefore, the surface air concentration on the source material, *y*_0_, remained constant with diffusion distance (S2(A) Fig). In contrast, when the emission rates from the source material do not change with diffusion distance, the rate-limiting step of DEHP emission from the source material is internal diffusion inside the material. In this case, the surface concentration on the source material, *y*_0_, decreased when the diffusion distance decreased because DEHP supply from the inside of the source material to the surface is insufficient compared to the demand (S2(B) Fig). Although PFS may create a different situation from actual environment, related to the thickness of the boundary layer and lack of convective mass transfer, PFS is adequate to evaluate the emission characteristics of emission source under controlled condition.

A polyethylene terephthalate (PET) resin was chosen to construct the body of the PFS apparatus based on the results of the phthalate leaching tests. These tests were conducted to assess which resin could contaminate due to leaching. No leaching was detected in the PET resin by acetone or dichloromethane extraction; however, leaching was detected from the other resins such as polycarbonate resin. A C18 (octadecyl) extraction disk (Empore SPE, 47 mm diameter, 3M, USA) was used as the adsorbent since glass fiber filter was inadequate as adsorbent due to the low sorption capability in the preliminary examination.

### Test samples

Two vinyl-flooring materials (denoted samples A (LG Hausys, Korea) and B (Fusogosei Co., Ltd., Japan)) and wallpaper (denoted sample C (Lilycolor Co., Ltd., Japan)) were used as the sample building materials. Sample A consist of surface PVC print layer, foamed PVC layer, and non-woven textile. Sample B consist of surface PVC print layer, foamed PVC layer, and non-woven textile. Sample C consist of surface PVC layer and paper lining. As shown in S3 Fig., the surface textures of samples A and B are smooth, while that of sample C is rough. The thicknesses of these samples are 1.75, 1.85, and 0.50 mm, respectively. The weight per unit area of these samples was 0.135, 0.0815, and 0.0251 g/cm^2^, respectively. The internal structures of samples A and B consist of multi-layer with a highly porous network (resembling that of Swiss cheese), while that of sample C is single-layer without holes (S3 Fig). Prior to sampling, the sample surfaces were wiped with a paper towel, wrapped with aluminum foil in plastic bag, and placed in a thermostatic chamber at 28ºC for 14 days.

### DEHP analysis

DEHP adsorbed onto the adsorbent was extracted in 3 mL dichloromethane using ultrasonication for 30 min, following which it was filtrated with a polypropylene filter (13CR; Nihon Pall Ltd., Japan). Then, extracted DEHP were analyzed using gas chromatography–mass spectrometry (GC-MS; Agilent 5973–6890, Agilent Technologies Inc., USA) equipped with a 5% phenylmethylsiloxane capillary column (30 m × 0.25 mm i.d. 0.25 μm film thickness, Agilent HP-5MS, Agilent Technologies, Inc, USA). The column temperature was maintained at 70°C for 2 min and then increased at a rate of 7°C/min to 280°C, where it was held for 1 min. The injection port and interface were kept at 250°C and 270°C, respectively. The injection volume was 1 μL and the splitless injection mode was used. Five concentrations of DEHP standard solution was re-determined every several samples. DEHP sampled with a porous polymer adsorbent (Tenax TA 60/80, Buchem B.V., The Netherlands) were thermally desorbed at 250°C and analyzed using GC-MS with the same method as solvent extraction.

### Contents of DEHP in the building materials

The contents of DEHP in the sample building materials were determined as follows: a piece of sample (0.5 g) was cut and dissolved in 20 mL of tetrahydrofuran (THF, HPLC grade, Wako Pure Chemical Industries, Ltd., Japan). One milliliter of dissolved solution was reprecipitated using methanol and diluted to 20 mL with methanol (HPLC grade, Wako Pure Chemical Industries, Ltd., Japan). One milliliter of the supernatant solution was diluted 10-times with methanol and analyzed with GC-MS.

### Determination of emission rates using PFS

Initially, total emission amounts from each building material were measured over sampling periods of 1, 3, 5, and 7 days at 2.5 mm of the diffusion distances for sample A and B and at 0.5 mm of the diffusion distances for sample C (depth of PFS minus the thickness of the adsorbent disk (0.5 mm)).

Next, emission rates from each building material were measured at different diffusion distances (0.5, 2.5, 5.0, and 7.5 mm) over a sampling period of 7 days. Each measurement was conducted at 28°C in a desiccator located in a thermostatic oven to prevent contamination. Since neither a temperature difference nor air-flow exists in the PFS, the diffusion distances can be considered as equal to the depth of PFS while disregarding the thickness of the adsorbent disk (0.5 mm). The emission rates were obtained by following equation.

$$E=\frac{M}{At}$$ (1)

where *E* is the emission rate [μg/m^2^/s], *M* is adsorbed amount in the adsorbent [μg], *A* is the area of adsorbent (= area of emission source) [m^2^], and *t* is sampling duration [s]

### Evaluation of emission characteristics

When the emission is limited by the diffusion in the boundary layer, the DEHP emission from the materials can be dependent on Fick’s Law, expressed as follows:

$$E=\frac{D_{air}(y_{0}-y_{ad})}{d}$$ (2)

where *E* is the emission rate [μg/m^2^/s], *D*_air_ is the molecular diffusion coefficient in air [m^2^/s], *y*_0_ is the surface-air concentration of DEHP on the building material [μg/m^3^], *y*_ad_ is the adsorbent surface-air concentration of DEHP [μg/m^3^], and *d* is the diffusion distance [m]. The adsorbent surface concentration, *y*_ad_, can be considered as 0 because of the strong adsorption of DEHP on the adsorbent disk (In the preliminary test, breakthrough to second disk was not observed in pump sampling). Thus, the emission rates can be expressed by the following equation:

$$E=D_{air}y_{0}\frac{1}{d}\;(\mathrm{when}\;y_{\mathrm{ad}}=0)$$ (3)

In addition, partition coefficient, *K*_material_air_ [unitless], can be expressed as follow,

$$K_{material\_air}=\frac{C_{0}}{y_{0}}$$ (4)

where *C*_0_ is the surface concentration of DEHP in the building material [μg/m^3^].

### Micro-chamber test

Emission rates from the building material samples were determined using the micro-chamber method (S4 Fig, GL Sciences Inc., Japan) according to ISO 16000–25 [19] and JIS A 1904 [25] specifications. In the method, the sample material is initially placed on the upper side of a 630 mL silane-treated glass micro-chamber at 28°C ± 1°C. Next, DEHP in the chamber air is collected with Tenax TA for 24 h at a chamber air-flow rate of 15 mL/min (air exchange rate: 1.43 /h). Then, after the sample material is removed from the micro-chamber, the adsorbed DEHP on the wall of the micro-chamber is desorbed at 250°C and collected with Tenax TA for 75 min at 60 mL/min (Table 1).

The micro-chamber interior air concentration can be written as

$$V\frac{dy_{chamber}}{dt}=E_{chamber}A_{emission}-S_{adsorption}A_{\mathrm{Sin}k}-Qy_{chamber}$$ (5)

where *V* is chamber volume [m^3^], *y*_chamber_ is the micro-chamber interior air concentration of DEHP [μg/m^3^], *E*_chamber_ is the emission rate from the sample in the chamber [μg/m^2^/s], *A*_emission_ is the area of the sample [m^2^], *S*_adsorption_ is the adsorption rate onto the chamber wall [μg/m^2^/s], *A*_sink_ is the area of the sample [m^2^], and *Q* is air exchange rates [m^3^/s].

In the steady state condition,

$$E_{chamber}A_{emission}=S_{adsorption}A_{\mathrm{Sin}k}+Qy_{chamber}$$ (6)

DEHP in the micro-chamber interior air, *Qy*_chamber_, can be sampled with 24-h sampling in the first step of micro-chamber. The adsorbed DEHP on the chamber wall, *S*_adsorption_*A*_sink_, can be collected by the 250°C desorption. Thus, the emission rates in the chamber, *E*_chamber_, can be obtained by the micro-chamber measurement.

In addition, the emission rate in the chamber, *E*_chamber_, can be written as

$$E_{chamber}=\frac{D_{air}}{d}\left(y_{0}-y_{chamber}\right)=h_{m}\left(y_{0}-y_{chamber}\right)$$ (7)

where *h*_m_ is the mass-transfer coefficient in the chamber [m/s]. *h*_m_ was calculated using obtained *E*_chamber_ and *y*_chamber_ obtained by the micro-chamber test and *y*_0_ obtained by the PFS test.

### Detection limit, recovery efficiency, precision, adsorption efficiency on the adsorbent, adsorption on the PFS wall

To investigate the detection limit, because DEHP was not detected in blank adsorbent disks (*N* = 5), the background signal/noise ratio was evaluated. To determine the recovery efficiency, aliquots of 100 μL of a dichloromethane solution of DEHP (1.0 μg per aliquot) were spiked onto five adsorbent disks, each in a separate Petri dish, with a microsyringe. The Petri dishes were then covered with tight-fitting glass plates for 60 min, during which time the solvent evaporated. To evaluate method precision (repeatability), side-by-side sampling of DEHP was carried out at five points on the same wallpaper material (Sample C) for 24 h at 0.5 mm of diffusion distance and for 7 days at 0.5 and 7.5 mm. To check the adsorption of the emitted DEHP on the PFS wall, DEHP amounts adsorbed onto the adsorbent disk and the PFS wall were determined at a diffusion distance of 7.5 mm for a sampling time of 7 days for Sample C (*N* = 5). In the test, adsorbed DEHP was wiped with piece of paper and extracted from the paper in 3 mL of dichloromethane using ultrasonication for 30 min.

To obtain the recovery efficiency for micro-chamber, 100 ng of DEHP was injected into micro-chamber through the uncoupled outlet line using micro syringe (1 μL) under pressurization with pure nitrogen followed by outlet line reconnection and purge of in-chamber air with pure nitrogen for a few minutes. After then, chamber was heated until 250°C and sampled with Tenax TA. To evaluate method precision (repeatability), emission rates from same sample (Sample A and C) were determined 5 times using micro-chamber.

## Results

### Determination of emission rates and estimation of surface concentrations using PFS

**Fig 3:**
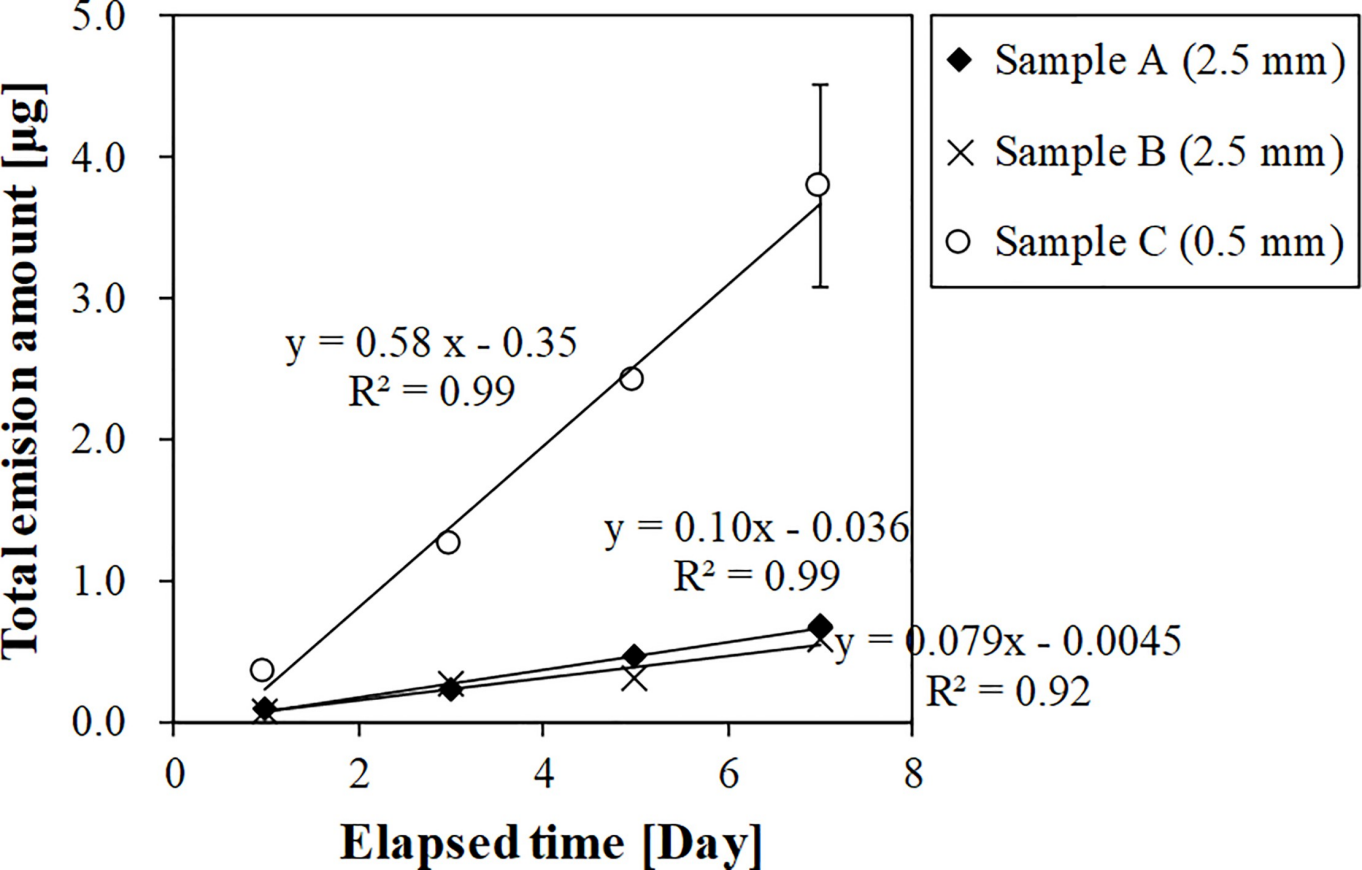
DEHP total emission amounts from samples A, B, and C over 7 days.

The trapped amounts of DEHP on the adsorbent disk with 0.5 or 2.5 mm of boundary thickness were proportional to the duration of the sampling periods (Fig 3, S1 Table). It was thus confirmed that the emission rate from these building materials remained constant at least during the 7-day sampling periods employed in this study.

**Fig 4:**
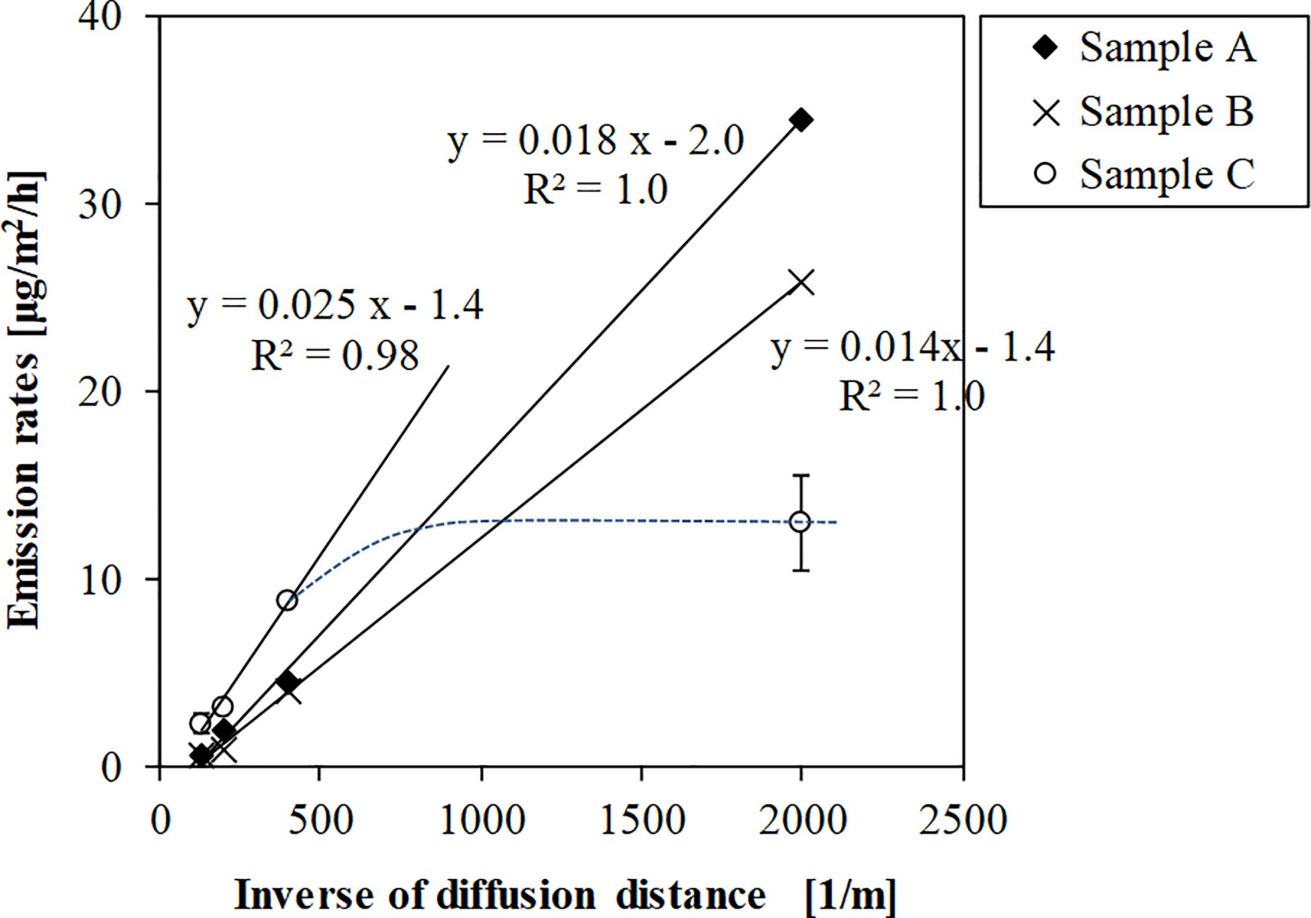
DEHP emission rates from samples A, B, and C measured using PFS vs. inverse of diffusion distance.

The dependency of DEHP emission rates on the diffusion distances inside the PFS is shown for the vinyl floorings and wallpaper in Fig 4 and S2 Table. The DEHP emission flux was proportional to the inverse of the diffusion distance inside the PFS between 0.5 and 7.5 mm for samples A and B, and between 2.5 and 7.5 mm for sample C (*R*^2^ = 0.999, 1.00, and 0.982 for samples A, B, and C, respectively). This result indicated that the DEHP emission from these materials is dependent on Fick’s Law. The intercept could indicate the DEHP adsorption on the wall of PFS. From the results, the emission flux was proportional to the inverse of the diffusion distance. The slopes of the regression line in Fig 4 corresponds to the value of *D*_air_*y*_0_; these observations imply that the vinyl flooring surface concentration, *y*_0_, remained almost constant with variations in the boundary layer thickness, since *D*_air_ is substance-specific and a constant parameter at the same temperature. The molecular diffusion coefficient of DEHP in air has been previously estimated as 2.6–3.9 × 10^−6^ m^2^/s according to the studies of Strommen and Kamens [26], Clausen et al. [27], and Liu et al. [28]. Hence, using 3.0 × 10^−6^ m^2^/s of the molecular diffusion coefficient, *y*_0_ was calculated as 1.7 and 1.3 μg/m^3^ for samples A and B, respectively, for a boundary layer of 0.5–7.5 mm. For sample C, *y*_0_ was calculated as 2.3 μg/m^3^ for a boundary layer of 2.5–7.5 mm, while that for a boundary layer of 0.5 mm decreased to 0.60 μg/m^3^.

### Determination of DEHP contents in the building materials and estimation of partition coefficient

The contents (weight concentrations) of DEHP contained in samples A, B, and C were 16.3%, 18.7%, and 15.4%, respectively. Since the weight per surface area of each material was 0.135, 0.0815, and 0.0251 g/cm^2^, DEHP concentrations per surface area were calculated as 2.2 × 10^8^, 3.9 × 10^8^, and 3.9 × 10^7^ μg/m^2^ and DEHP concentrations per unit volume, *C*_0_, were calculated as 1.3 × 10^11^, 8.2 × 10^10^, and 7.7 × 10^10^ μg/m^3^, respectively. If the DEHP contents in these samples are uniformly distributed across the depth, the surface concentration in the material could be considered as the DEHP contents per unit volume. By using the Eq (4), partition coefficient, *K*_material_air_ were calculated to be 7.5 × 10^10^, 6.6 × 10^10^, and 3.3 × 10^10^, for samples A, B, and C, respectively. For 0.5 mm of boundary layer thickness on sample C, *C*_0_ of the surface could be decrease to 2.0 × 10^10^ μg/m^3^ because rate-limiting step was changed [13].

### Estimation of boundary layer thickness and mass-transfer coefficients in micro-chamber

DEHP concentrations adsorbed onto and thermally desorbed from the micro-chamber wall could be determined, although the micro-chamber interior air concentration of DEHP was under the detection limit for all the samples. The emission rates determined using micro-chamber analyses were 6.0, 4.5, and 6.1 μg/m^2^/h for sample A, B, and C, respectively (S3 Table). Since the micro-chamber interior air concentration of DEHP was lower than the detection limit, half of detection limit was used as the micro-chamber interior air concentration of DEHP, *y*_chamber_ (0.23 μg/m^3^). In addition, *y*_0_ could be equal between PFS and micro-chamber because the rate-limiting step of DEHP emission was the diffusion in the gas-phase boundary layer. Therefore, *y*_0_ obtained in PFS test were used for the calculation. Hence, the mass-transfer coefficients, *h*_m_, in the micro-chamber were calculated as 1.1 × 10^−3^ and 1.2 × 10^−3^ m/s for samples A and B, and 8.1 × 10^−4^ m/s for sample C. In addition, the thicknesses of boundary layer were estimated to be 2.6 × 10^−3^, 2.5 × 10^−3^, 3.7 × 10^−3^ m, respectively, by using 3.0 × 10^−6^ m^2^/s as the molecular diffusion coefficient according to the previous studies, 2.6–3.9 × 10^−6^ m^2^/s.

### Detection limit, recovery efficiency, and precision, and uncertainty

The detection and determination limits for DEHP, estimated from the respective signal/noise ratios of 3 and 10, were calculated as approximately 3 and 10 ng, respectively, for a single filter. Hence, the lower determination limits for DEHP emission flux using PFS were calculated as 0.24 and 0.034 μg/m^2^/h for 24-hour and 7-day sampling periods, respectively. The recovery efficiency of spiked DEHP (1 μg; 100 μL; *N* = 5) from adsorbent disk using in PFS was 98% ± 10%. The method precisions of the emission rate measurement with PFS were 8.0% for 24 h sampling at 0.5 mm of diffusion distance (*N* = 5), and 19% and 21% for 7 days sampling at 0.5 and 7.5 mm of diffusion distance (*N* = 5). Adsorbed amounts on the adsorbent disk and PFS wall (7.5 mm) during 7 days sampling were 1.1 ± 0.3 μg (*N* = 5) and 1.1 ± 0.2 μg (*N* = 5), respectively.

Since the determination limits for DEHP were approximately 10 ng, the lower determination limits for DEHP emission flux using micro-chamber were calculated as 0.016 μg/m^2^/h for 24-hour sampling periods. The recovery efficiency of spiked DEHP (100 ng; 100 μL; *N* = 5) from micro-chamber was 94% ± 8.8%. The method precisions of the emission rate measurement with micro-chamber were 4.1% (*N* = 5) and 5.8% (*N* = 5) for sample A and C, respectively.

The molecular diffusion coefficient of DEHP in air has been previously estimated as 2.6–3.9 × 10^−6^ m^2^/s. We use a value of 3.0 × 10^−6^ m^2^/s for the molecular diffusion coefficient. Here, the error was ~30%. In the PFS analysis for a sampling time of 7 days, the precision value was ~21%. Considering the adsorption on the PFS wall, *y*_0_ could increase by 27% (described in Discussion). Precision value for the resin contents test and micro-chamber test were ~10% [29] and 4.1–5.8% [30], respectively. Therefore, the total uncertainty, which can be calculated using the root sum square of each error, could be up to 45% for the surface concentration, *y*_0_, 47% for the partition coefficient, *K*_material_air_, and 46% for the mass transfer coefficient in micro-chamber, *h*_m_. Most of the uncertainty arises from the literature data of molecular diffusion coefficient.

## Discussion

The emission rates were observed to be constant over the week-long sampling period. The time at which it reaches equilibrium, indicated from intercept, were 1.4–15 hours. The time required to obtain the constant emission rates for PFS analysis is shorter than those of most other methods to measure DEHP emission rates (FLEC: 10–20 days at 23°C [15], ca. 100 days [13], over 150 days [12]; CLIMPAQ: ca. 150 days [13], over 150 days [12], sandwich chamber: 20 days [2]) owing to the larger surface adsorption area of the instruments, although recently developed method realize the shorter test duration to 2–5 days [14] and less than 24 hours [24].

The emission flux was linearly related to the inverse of diffusion distance (Fig 4). The regression line, however, had negative intercept; this result could be attributed to the loss of diffused DEHP due to the sorption on the PET wall of the PFS. The results show that the adsorbed amounts on the PFS wall (1.1 ± 0.2 μg) were identical to the sampling amount in the adsorbent disk (1.1 ± 0.2 μg). Assuming the emission from the outer toric edge, whose width is the same as the diffusion distance, was adsorbed on the PFS wall, the underestimation was 4%, 20%, 39%, and 54% at diffusion distances of 0.5, 2.5, 5.0, and 7.5 mm, respectively. If the measured value was corrected for the underestimation, the surface air concentration, *y*_0_, could be underestimated by 5.1%, 5.2%, and 27% for samples A, B, and C, respectively. According to Cao et al., in which the adsorption on the wall can be negligible when the ratio of wall area to emission area was <0.1 [21], wall adsorption only for 0.5 mm of diffusion distances can be negligible. For the future research, a less adsorbent material instead of PET might be preferable for the PFS body.

The emission rates from the vinyl floorings (samples A and B) were dependent on Fick’s law, indicating that the rate-limiting step of DEHP emission from the vinyl floorings was the diffusion in the gas-phase boundary layer and/or convective transfer in the indoor environment. In these cases, the demand is insufficient compared to the supply capacity of DEHP from the inside to the surface of the building materials. Therefore, the surface air concentration on vinyl floorings, *y*_0_, remained constant with the diffusion distance (S5 Fig). In contrast, the emission rates from the wallpaper (sample C) were not dependent on Fick’s law at a diffusion distance less than 2.5 mm, indicating that DEHP emission from the wallpaper was likely to be controlled by both the diffusion through the air boundary layer and the internal diffusion inside the material. In this case, the DEHP demand is higher than the supply from the inside to the surface of the wallpaper. Therefore, surface concentration on the wallpaper, *y*_0_, decreased when the diffusion distance decreased (S5 Fig). From these results, it can be inferred that the emission rates are dependent on air-flow velocities for a building material whose emission is limited by gas-phase diffusion in the boundary layer and/or convective transfer. Different ventilation strategies depending on the building materials can be implemented in the indoor environment.

Despite the low vapor pressure and low saturated concentrations (relative to VOCs) of phthalate, the vinyl flooring surface concentrations of DEHP (*y*_0_ = 1.7, 1.3, and 2.3/0.60 μg/m^3^ for samples A, B, and C, respectively) were lower than the estimated saturated vapor concentrations of DEHP at 25°C (5.2 μg/m^3^ (3.04 × 10^−5^ Pa) [31]). The material surface-air concentrations, *y*_0_, described here are similar to those levels reported for several kinds of PVC floorings: 0.9 and 1.0 μg/m^3^ at 23°C [15]; ca. 1.6–2.7 μg/m^3^ at 20°C [16]; 1.1 μg/m^3^ [2, 13] because the saturated concentration is within a factor of 2 between 23ºC and 28ºC. Since the surface-air concentration is dependent on the sample DEHP content and structure, an equilibrium is established between the surface-air and in-sample concentrations as represented by the partition coefficient [16, 32]. In the present study, the partition coefficients were estimated to be 3.3–7.5 × 10^10^, and these partition coefficients were similar range to those estimated for particles (10^8^–10^13^) [32] in the previous study [33]. However, the actual DEHP surface concentrations, *C*_0_, in the sample materials could be much higher than those estimated in the present study because the samples consist of thin vinyl chloride film on the upper side, within which most of the DEHP was present, and an adhesive/mat board on the underside, which contained much lesser amounts of DEHP.

DEHP emission from these building materials were also determined using micro-chamber. Mass-transfer coefficient in the micro-chamber were estimated from the results. The measured mass-transfer coefficients, *h*_m_, 0.81–1.2 × 10^−3^ m/s, were larger than those obtained in CLIMPAQ (4.0 × 10^−4^ m/s) [13], sandwich chamber (4.0 × 10^−4^ m/s) [2], airtight chamber (2.4 × 10^−5^ m/s) [34], and 20-L chamber (7.2 × 10^−4^ m/s) [35], and were smaller than those obtained in FLEC (1.4 × 10^−3^ m/s) [13]. Since air flows in the vertical direction exist in the micro-chamber unlike CLIMPAQ and the sandwich chamber, the thickness of the boundary layer in the micro-chamber could be thinner than that in CLIMPAQ and the sandwich chamber. This could be one reason for the large mass-transfer coefficients. The thickness of the surface boundary layer is quite thin and the mass-transfer coefficient is large because FLEC has a high air flow. The difference in mass-transfer coefficient can be attributed to the difference in surface flow velocity.

DEHP transfer was conducted by the molecular diffusion in the PFS, while DEHP transfer was conducted by the molecular diffusion, convective flow due to the ventilation, and the turbulence diffusion in the micro chamber. Assuming interior of micro-chamber could be virtually-divided to the boundary layer, in which only molecular diffusion affect to the transfer, and bulk space, in which convective flow and turbulent flow affect to the transfer, the boundary layer thickness in the micro-chamber was estimated by dividing mass transfer coefficient by molecular diffusion coefficient. The thickness was calculated to be 2.6 and 2.5 mm for samples A and B, and 3.7 mm for sample C. This could be because the surface convective flow velocity of sample C, which has rough surface texture, could be lower than those of samples A and B, which have smooth surface texture.

The advantages of PFS are short determination period, possibility of field sampling, and low cost, while disadvantages attributed to PFS include poor sample representation owing to the small sampling area as compared to FLEC, CLIMPAQ, and sandwich chamber methods, which can be used for larger samples. However, the variability of DEHP emission from vinyl flooring can be quite small [16]; because DEHP is added evenly and in excess to resins as a plasticizer, the resin structure remains uniform on the macro level. Therefore, PFS can be useful for the measurement of plasticizers in building materials.

The emission rates measured by PFS and micro-chamber analysis could in fact be overestimations as compared with an actual indoor environment, since the concentration gradient in the boundary layer in the PFS or micro-chamber is larger than that in an indoor environment (ca. 0 μg/m^3^ of surface concentration on the adsorbent or in-chamber concentration). The emission rate in an indoor environment, *E*_indoor_ [μg/m^2^/s], can be expressed as follows:

$$E_{indoor}=h_{m,indoor}(y_{0}-y)$$ (8)

where *y* is indoor concentration [μg/m^3^] and *h*_m,indoor_ is the mass-transfer coefficient in the real room [m/s]. For samples A, B, and C, the interior surface-air concentration, *y*_0_, was considered to remain constant with different indoor concentrations as described above when the indoor concentration was lower than the surface concentration. A DEHP indoor concentration of 0.012–1.7 μg/m^3^ (median: 0.15 μg/m^3^) as found in Japanese residential homes [4] was supposed. Assuming that the mass-transfer coefficient, *h*_m,indoor_ is 1/3 of the value obtained in the micro-chamber, the emission rates in a general indoor environment (median), *E*_indoor_, were calculated as 0.0–2.3, 0.0–1.8, and 0.61–2.2 μg/m^2^/h for samples A, B, and C, respectively. In an indoor environment where other high-emission sources might be present and indoor concentration is high, samples A and B may not be emission sources of DEHP but could rather act as indoor DEHP sinks.

## Conclusion

In this study, the emission rates of DEHP from three building material samples—two types of vinyl floorings and one type of wallpaper—were determined using PFS and micro-chamber analytical techniques. DEHP emission rates remained unchanged for at least one week after the start of PFS sampling, indicating little adsorption onto the PFS itself. The emission rates from the vinyl flooring materials measured at different diffusion distances (0.5, 2.5, 5.0, and 7.5 mm) were linearly related to the inverse of the diffusion distances, indicating in-boundary layer diffusion rate-limiting step for emission from these materials. Further laboratory tests were performed to apply the emission rate data obtained using PFS to estimate boundary layer thickness in micro-chamber. From these results, the boundary layer thicknesses on the building materials in the micro-chamber were estimated to be 2.5–3.7 mm for the vinyl floorings and wallpaper, respectively.

## Supporting information

S1 Table Raw data of total emission amount during 7 days. (PDF)

S2 Table Raw data of emission rates for different boundary thickness. (PDF)

S3 Table Raw data of emission rates in the micro-chamber test. (PDF)

S1 Fig Schematic representation of semi-volatile organic compounds (SVOCs) in an indoor environment (Detailed version). (PDF)

S2 Fig Schematic diagram of the relationship between boundary layer air concentration and boundary layer thickness. (A) In-boundary layer diffusion limiting and (B) Inside-material diffusion (supply to surface) limiting.
(PDF)

S3 Fig Photographs of the upper side, lower side, and cross-section of samples A, B, and C.
(PDF)

S4 Fig (A) Photographs of micro-chamber and (B) diagram illustrating design of micro-chamber.
(PDF)

S5 Fig Schematic diagram of surface concentration and concentration gradient for sample A, B, and C.
(PDF)
