# Supplementary material for: Emission characteristics of diethylhexyl phthalate (DEHP) from building materials determined using a passive flux sampler and micro-chamber
Source: PLoS One. 2019 Sep 20;14(9):e0222557. doi: 10.1371/journal.pone.0222557 (PMC6754160; doi:10.1371/journal.pone.0222557)
Supplement: S1 Table — (PDF) [file pone.0222557.s001.pdf]

S1 Table. Raw data of total emission amount during 7 days.

	Elapsed time	1 day	3 day	5 day	7 day
Total emission amount [μg]	Sample A	0.088	0.23	0.47	0.68
	Sample B	0.081	0.27	0.30	0.59
	Sample C	0.35 ± 0.028	1.3	2.4	3.8 ± 0.72
