# Supplementary material for: Emission characteristics of diethylhexyl phthalate (DEHP) from building materials determined using a passive flux sampler and micro-chamber
Source: PLoS One. 2019 Sep 20;14(9):e0222557. doi: 10.1371/journal.pone.0222557 (PMC6754160; doi:10.1371/journal.pone.0222557)
Supplement: S2 Table — (PDF) [file pone.0222557.s002.pdf]

S2 Table. Raw data of emission rates for different boundary thickness.

	Inverse of boundary thickness [1/m]	2000	400	200	133
	Boundary thickness [mm]	0.50	2.5	5.0	7.5
Emission rate [μg/m^2/h]	Sample A	34	4.5	2.0	0.70
	Sample B	26	4.1	0.97	0.62
	Sample C	13 ± 2.5	8.8	3.1	2.4 ± 0.49
