# Supplementary material for: Emission characteristics of diethylhexyl phthalate (DEHP) from building materials determined using a passive flux sampler and micro-chamber
Source: PLoS One. 2019 Sep 20;14(9):e0222557. doi: 10.1371/journal.pone.0222557 (PMC6754160; doi:10.1371/journal.pone.0222557)
Supplement: S3 Table — (PDF) [file pone.0222557.s003.pdf]

S3 Table. Raw data of emission rates in the micro-chamber test.

	Emission rate [μg/m^2/h]
Sample A	6.0 ± 0.25
Sample B	4.5
Sample C	6.1 ± 0.35
