# Supplementary material for: Emission characteristics of diethylhexyl phthalate (DEHP) from building materials determined using a passive flux sampler and micro-chamber
Source: PLoS One. 2019 Sep 20;14(9):e0222557. doi: 10.1371/journal.pone.0222557 (PMC6754160; doi:10.1371/journal.pone.0222557)
Supplement: S1 Fig — (PDF) [file pone.0222557.s004.pdf]

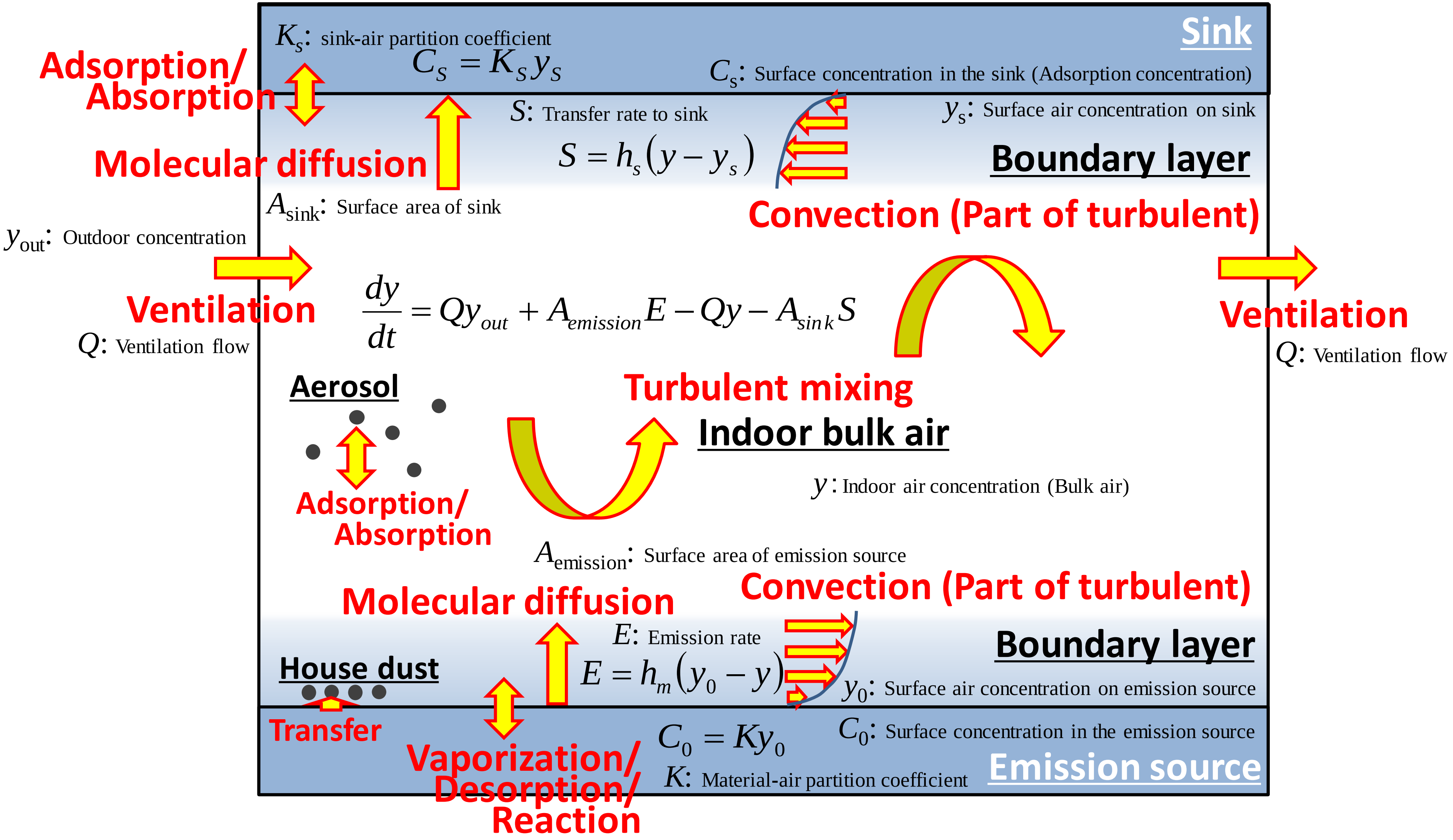

S1 Figure. Schematic representation of semi-volatile organic compounds (SVOCs) in an indoor environment (Detailed version).
