# Supplementary material for: Emission characteristics of diethylhexyl phthalate (DEHP) from building materials determined using a passive flux sampler and micro-chamber
Source: PLoS One. 2019 Sep 20;14(9):e0222557. doi: 10.1371/journal.pone.0222557 (PMC6754160; doi:10.1371/journal.pone.0222557)
Supplement: S2 Fig — (A) In-boundary layer diffusion limiting and (B) Inside-material diffusion (supply to surface) limiting. (PDF) [file pone.0222557.s005.pdf]

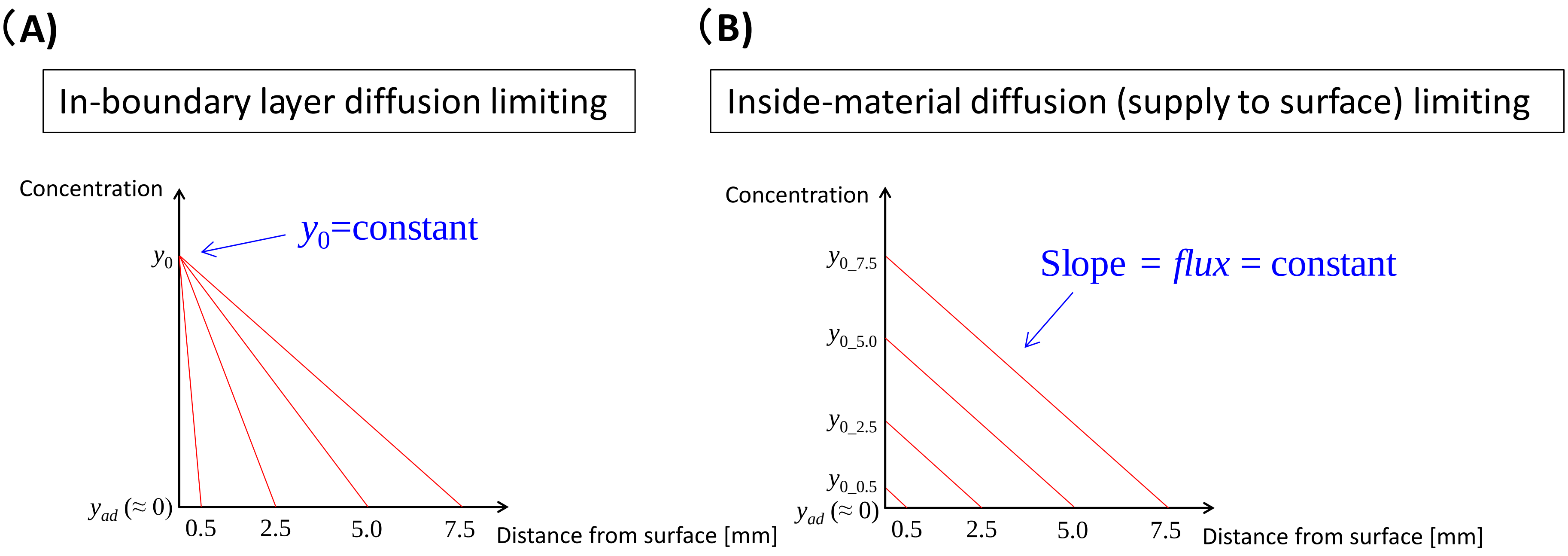

S2 Figure. Schematic diagram of the relationship between boundary layer air concentration and boundary layer thickness. (A) In-boundary layer diffusion limiting and (B) Inside-material diffusion (supply to surface) limiting.
