# Supplementary figures and images for: Emission characteristics of diethylhexyl phthalate (DEHP) from building materials determined using a passive flux sampler and micro-chamber
Source: PLoS One. 2019 Sep 20;14(9):e0222557. doi: 10.1371/journal.pone.0222557 (PMC6754160; doi:10.1371/journal.pone.0222557)

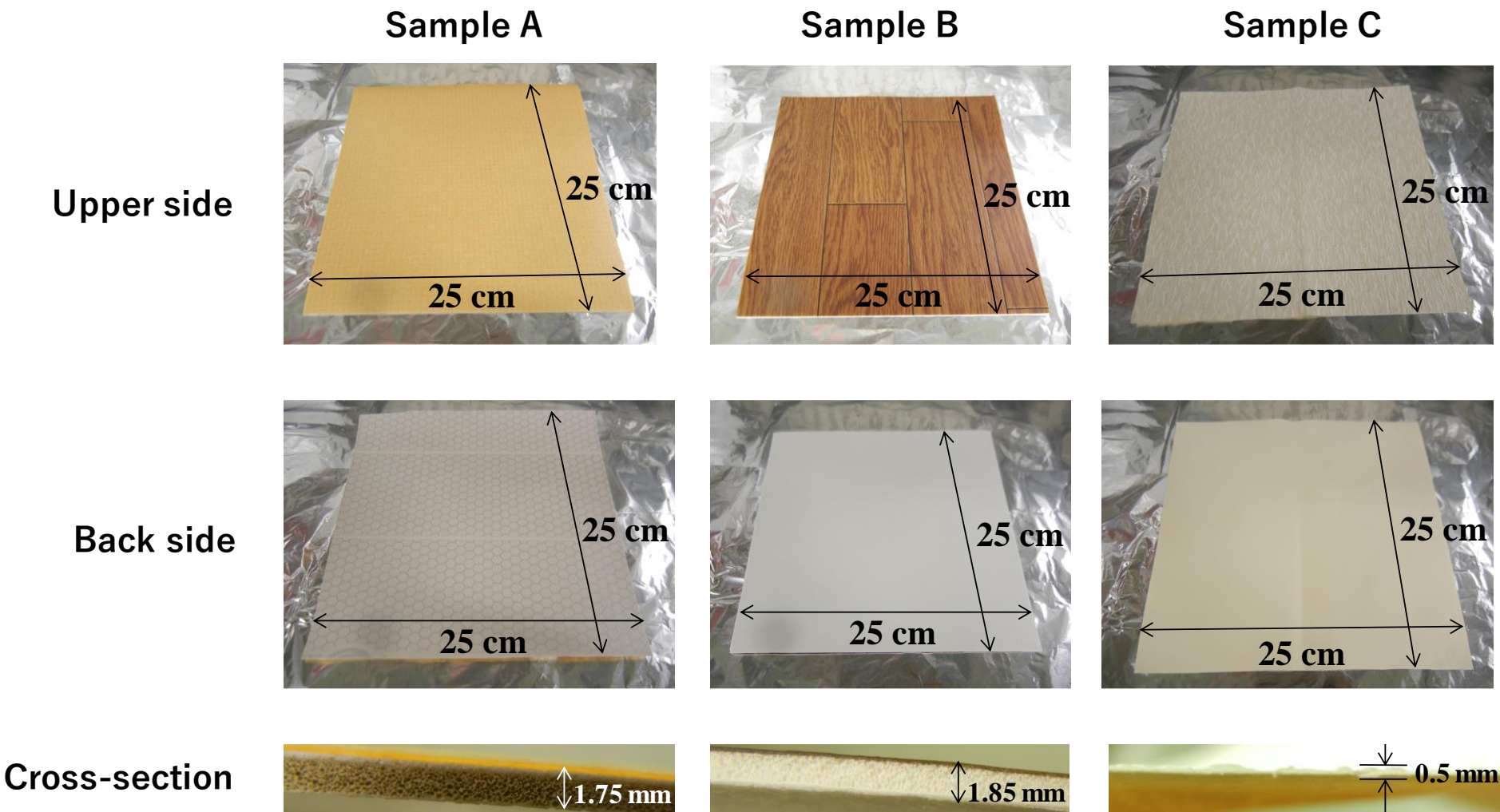

**S3 Figure.** Photographs of the upper side, lower side, and cross-section of samples A, B, and C.

Supplement: S3 Fig — Photographs of the upper side, lower side, and cross-section of samples A, B, and C. (PDF) [file pone.0222557.s006.pdf]

(A)

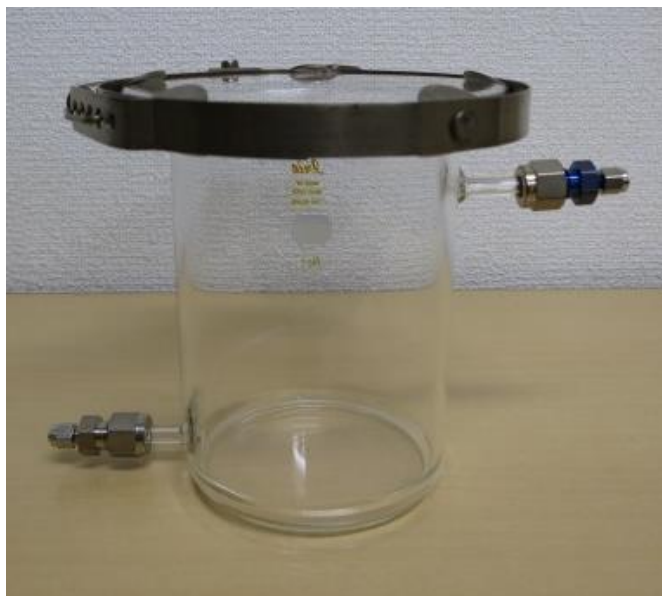

(B)

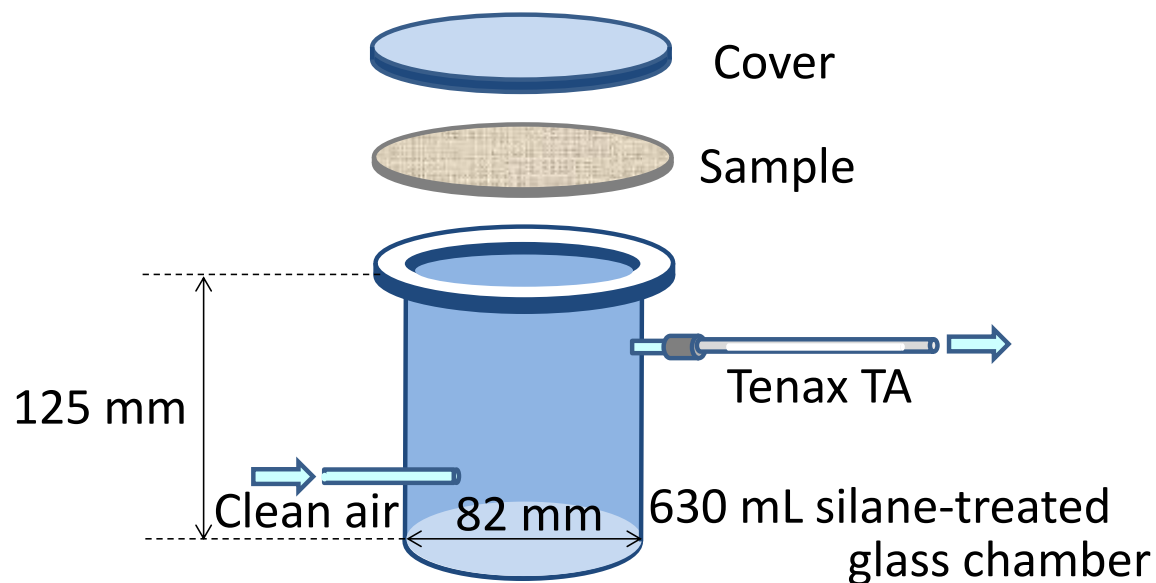

**S4 Figure.** (A) Photographs of microchamber and (B) diagram illustrating design of micro-chamber.

Supplement: S4 Fig — (A) Photographs of micro-chamber and (B) diagram illustrating design of micro-chamber. (PDF) [file pone.0222557.s007.pdf]
