# Supplementary material for: Emission characteristics of diethylhexyl phthalate (DEHP) from building materials determined using a passive flux sampler and micro-chamber
Source: PLoS One. 2019 Sep 20;14(9):e0222557. doi: 10.1371/journal.pone.0222557 (PMC6754160; doi:10.1371/journal.pone.0222557)
Supplement: S5 Fig — Schematic diagram of surface concentration and concentration gradient for sample A, B, and C. (PDF) [file pone.0222557.s008.pdf]

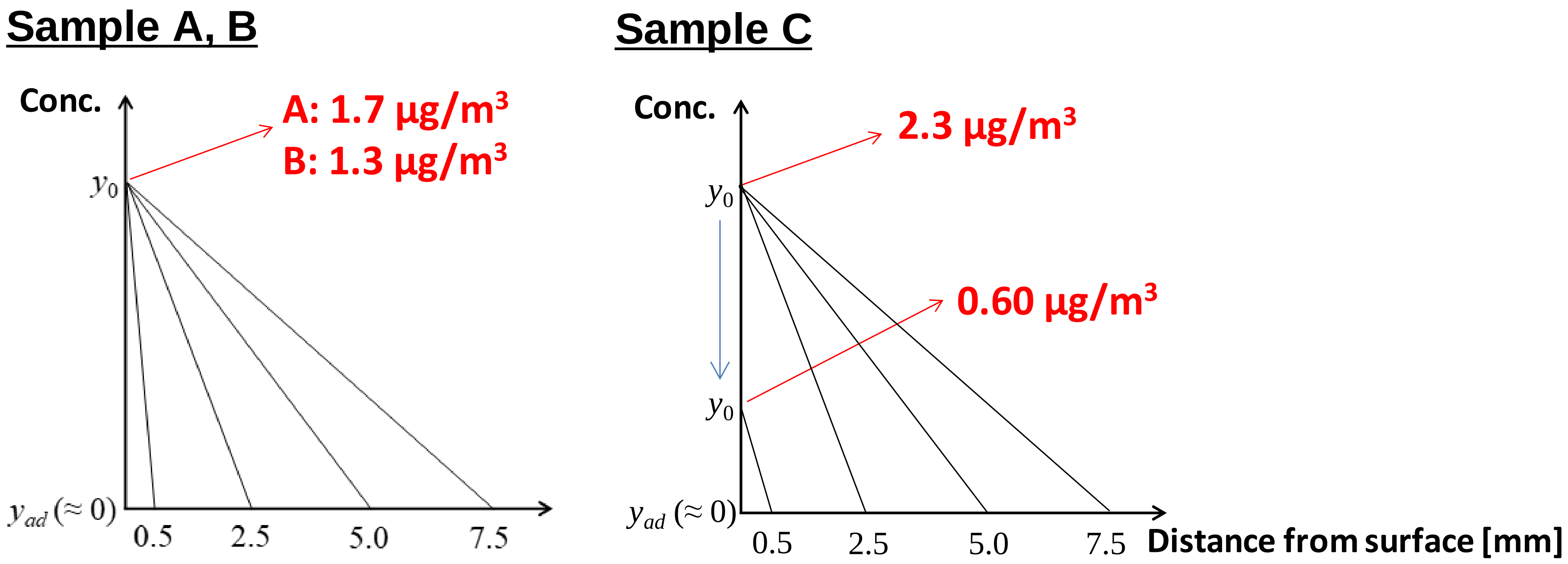

S5 Figure. Schematic diagram of surface concentration and concentration gradient for samples A, B, and C.
